# Stress response and virulence factors in bacterial pathogens relevant for Chilean aquaculture: current status and outlook of our knowledge

**DOI:** 10.1186/s40659-022-00391-5

**Published:** 2022-05-31

**Authors:** Derie E. Fuentes, Lillian G. Acuña, Iván L. Calderón

**Affiliations:** 1 Aquaculture and Marine Ecosystems, Center For Systems Biotechnology, Fraunhofer Chile Research, Santiago, Chile; 2 Environmental Sustainability, Center for Systems Biotechnology (CSB-UNAB), Universidad Andres Bello, Facultad de Ciencias de la Vida, Santiago, Chile; 3 Laboratorio de RNAs Bacterianos, Departamento de Ciencias Biológicas, Facultad de Ciencias de la Vida, Universidad Andres Bello, Santiago, Chile; 4 Interdisciplinary Center for Aquaculture Research, Universidad Andres Bello, Viña del Mar, Santiago, Chile

**Keywords:** Bacterial stress response, Oxidative stress, Iron metabolism, Virulence factors, *Piscirickettsia salmonis*, *Renibacterium salmoninarum*, *Tenacibaculum spp*, *Yersinia ruckeri*

## Abstract

The study of the stress responses in bacteria has given us a wealth of information regarding the mechanisms employed by these bacteria in aggressive or even non-optimal living conditions. This information has been applied by several researchers to identify molecular targets related to pathogeny, virulence, and survival, among others, and to design new prophylactic or therapeutic strategies against them. In this study, our knowledge of these mechanisms has been summarized with emphasis on some aquatic pathogenic bacteria of relevance to the health and productive aspects of Chilean salmon farming (*Piscirickettsia salmonis, Tenacibaculum spp., Renibacterium salmoninarum,* and *Yersinia ruckeri*). This study will aid further investigations aimed at shedding more light on possible lines of action for these pathogens in the coming years.

## Introduction

According to the Chilean authority, Sernapesca, salmonid species such as the Atlantic salmon (Salmo salar), rainbow trout (Oncorhynchus mykiss), and coho salmon (Oncorhynchus kisutch) have dominated aquaculture in Chile for the past 40 years, accounting for 72 percent, 8%, and 20% of current cultivation centers, respectively [1]. This production activity has grown due to the increasing and continuous need for healthy proteins and the interest of developed markets for high-quality salmon fillets, increasing from 100 thousand tons at the end of the 90 s to over 800 thousand tons in 2019—2020 [1]. However, rapid production growth has created favorable conditions for the emergence of infectious diseases which pose a threat to the sustainability of the industry, not only through mortality and decreased productivity, but also through environmental factors, such as the widespread use of antibiotics and pesticides which affect the industry’s image and access to markets [2, 3].

Some of these diseases are caused by bacterial pathogens, such as *P. salmonis*, *R. salmoninarum*, *Tenacibaculum spp.*, and *Y. ruckeri* among others. These pathogens vary greatly in their current economic relevance to the industry, and the research interest they have generated in recent years, but they all share the lack of effective control methods [2, 4].

In the first place, *P. salmonis* is a Gram-negative, non-motile, pleomorphic, and facultative intracellular Gammaproteobacteria [5]. It is the most important bacterial pathogen for salmon farming in Chile causing salmonid rickettsial septicemia (SRS), which accounts for about 55% of infectious disease fatalities in *Salmo salar,* 73% in *O. mykiss*, and about 60% in *O. kisutch* [1].

*Tenacibaculum* spp., on the other hand, are Gram-negative bacteria that are morphologically observed as straight filamentous rods [6]. They are responsible for an ulcerative disease known as marine tenacibaculosis that presents with ulcers with yellow plaques around the mouth and on epidermal surfaces of fish [7–11]. Some *Tenacibaculum* species are currently considered emerging pathogens for salmonid cultures in other salmonid-producing countries [9, 12]. It is important to note, however, that this genre is considered the second most relevant bacterial pathogen for Chilean salmon farms, with about 30% of mortalities related to infectious diseases in *S. salar,* and about 10% of mortalities in *O. mykyss* and *O. kisutch* in the year 2020 alone [1].

The third relevant pathogen is *R. salmoninarum*. It is the oldest known bacterial pathogen of fish and the only gram-positive bacterium on this list. It is a non-motile, non-capsulated, non-endospore forming, aerobic bacteria that causes bacterial kidney disease in salmonids [13]. Frequently, this pathogen produces a chronic infection in fish at low temperatures [14], accounting for about 9,5% of mortalities related to infectious diseases in *S. salar*, about 5% of mortalities in *O. mykiss,* and about 20% of mortalities in *O. kisutch* in Chile in 2020 [14].

Finally, *Y. ruckeri* is a facultative, Gram-negative pathogen that causes enteric redmouth disease (ERM), a septicemic disease that primarily affects salmonids, and on the large scale, the global fish farming industry [15]. In recent years, the number of outbreaks provoked by this pathogen in the aquaculture industry has substantially increased, affecting fish at all stages of development, and exhibiting higher mortality in adult specimens [16]. Since this disease has been unknown in Chile, there are no epidemiological records regarding its prevalence and distribution, which is expected to change in the coming years, given the global increase in cases.

All of these pathologies currently lack highly effective treatments and preventive tools to prevent infections at a high rate, as evidenced by the annual prevalence of some of them [2–5]. This is due in part to the fact that Chilean aquaculture and research initiatives to address its health problems are only 20 years old. However, there is increasing interest in the exhaustive study of the causal agents of these diseases, which has increased the volume of studies in this area. For example, commercially available vaccines to control "piscirickettsiosis" (the generic name for acute infection with *P. salmonis*) were tested a few years ago [2] providing a good overview of their characteristics, and providing development proposal for new and better alternatives, focusing primarily on the pathogen-host response analysis, and the use of surface proteins. Another recent study looked at the current status of piscirickettsiosis research, and the gaps that still need to be filled [17]. As a general observation, the majority of the research done thus far with these pathogens has been concentrated on epidemiological, pharmacological, and genomic fields, with very few, or almost none of them focusing on the biochemical or mechanistic components of their virulence repertoires. In this way, analyzing and understanding what gaps need to be filled in this specific field not only opens possibilities to develop better and more effective treatments and preventive options but also paves way for future research lines for our young scientists.

## Main text

Both prokaryotic and eukaryotic cells rely on homeostasis to function properly. For this reason, some organisms in nature have evolved a series of metabolic mechanisms aimed at reducing the damage generated by different types of stress. Pathogenic bacteria, for example, must frequently tolerate adverse conditions both outside and inside their respective hosts. For example, the evolution of adaptation and persistence mechanisms to withstand the specific conditions of the respiratory and gastrointestinal systems [18, 19]. Many of these factors are also involved in pathogenesis, virulence, and even antibiotic tolerance, which have been extensively studied and described in several bacterial pathogens of animals and humans, such as *Salmonella enterica*, *Shigella* spp, *Escherichia coli*, *Helicobacter* spp, *Vibrio* spp, *Campylobacter jejuni*, among several others [18–21]. The study of the expression and influence of these factors in these pathogenic models has led to the rational design of effective control mechanisms and treatment strategies for the associated diseases. Similarly, reviewing how much we know about these mechanisms in pathogens could be a useful strategy for the design of better control mechanisms soon.

## Oxidative stress response

The ability of some of these specific bacteria to thrive inside target cells makes them difficult to deal with. *P. salmonis,* for instance, is a facultative intracellular pathogen that invades the salmon’s liver, kidney, spleen, intestine, brain, ovary, and gills [5]. Furthermore, *P. salmonis, Y. ruckeri,* and *R. salmoninarum* can survive and reproduce in salmon macrophages and monocytes [22–29]. This very fact explains their ability to evade and/or resist the oxidative stress associated with this condition. In this regard, some investigations have revealed that rainbow trouts injected with *R. salmoninarum* produced more Reactive Nitrogen Species (RNS), such as NO measured in the serum, as well as in the gills and kidney tissues of those fish [30], thus suggesting that RNS play an important role in the defense against this pathogen.

The detoxifying capacity of host organisms against reactive oxygen species (ROS) and nitrogen (RNS) is widely distributed in nature and is related to the expression of a set of specialized enzymes such as catalases and superoxide dismutases, among others, previously identified in a variety of microorganisms, including well-known pathogens such as Salmonella, and also genetically related bacteria such as *Francisella* spp [18, 19, 21, 31–34].

In this regard, genomic and genetic analyses of virulent strains of *P. salmonis* have enabled the identification of several genes with high homology to others previously associated with stress response and virulence mechanisms [35–41], such as ROS and RNS detoxification systems, toxin-antitoxin systems, iron capture, and transport systems, porins and channels, chaperon proteins, and others. Similar bioinformatic analyses have revealed that *Tenacibaculum maritimum* genome encodes three superoxide dismutases: manganese-dependent SodA, iron-dependent SodB, and zinc-dependent SodC superoxide dismutases [42], as well as two catalase/peroxidase (KatA and KatG), indicating a strong and sophisticated mechanism to deal with oxidative stress, which is supported by the fact that treatments with hydrogen peroxide did not significantly reduce *T. maritimum* infections in fish [43]. Other investigations have demonstrated that several genes related to heavy metal resistance, such as the drug efflux system AcrA–AcrB–TolC from *E. coli*, can remove cationic heavy metals and limit ROS generation in *Tenacibaculum* [44, 45].

In the instance of *R. salmoninarum*, a bioinformatic study of its genome identified genes involved in oxidative stress response, as well as genes involved in virulence and antibiotic-resistance strategies with higher similarities to *Mycobacterium* [46]. A 57-kDa protein (p57) found on the bacterial cell surface which can be secreted in host environments [47], thus acting as a potent inhibitor of the respiratory burst is another critical component for oxidative stress response and virulence in *R. salmoninarum* [30, 48]. Furthermore, p57 is also linked to autoaggregation and virulence of this pathogen [49, 50]. With regards to the oxidative stress response, SodA plays a vital role during *R. salmoninarum* infection by catalyzing the conversion of superoxide anions to hydrogen peroxide [46].

The genes *sodA* and *sodB,* which encode manganese-dependent and iron-dependent superoxide dismutases, respectively, were also found in the *Y. ruckeri* genome [51] confirming its widespread distribution. Interestingly, the existence of small non-coding RNAs homologous to RyhB (called RhyB-1 and RhyB-2) have been described in *Y. ruckeri*, which are produced in salmon cells under iron deprivation circumstances to suppress the development of *sodB* [52]. This could allow the pathogen to tailor the relative levels of SodA and SodB isozymes to its nutritional and growth requirements. This is especially beneficial when iron concentrations are low and antioxidant enzymes are required (for instance, inside the host).

Another study considered the existence of small RNAs (sRNAs) in the genome of *P. salmonis*, several of which are linked to stress responses and the development of virulence factors [53]. The t44 sRNA was previously linked to early phases of *Salmonella* Typhimurium infection, and the Sau-5971 and RsaA sRNAs were linked to the potential of *Staphylococcus aureus* to generate resistant phenotypes to various unfavorable environmental conditions, including antibiotic treatments [53].

As a result, the link between the general metabolism of the bacteria and the regulation of the stress response is more complex. There is a direct relationship between the induction of the oxidative response mediated by ROS and/or RNS and the stringent conditions that bacteria face during the infective process, as well as the expression of factors associated with tolerance/resistance to antibiotics, increased virulence, and tolerance to vital nutrient limitations, such as iron and amino acids [54–58], which has previously been characterized in several pathogens such as *E. coli*, *M. tuberculosis,* and *P. aeruginosa*, among others [18, 19, 21].

Pulgar et al*.* (2015), for instance, announced the identification of 143 putative virulence genes distinct from those previously identified as siderophores and OMPs within the genome of *P. salmonis* LF89 strain [37], emphasizing the presence of the core genes of the type IV secretion system (T4SS), which had already been identified SPS:refid::bib36(36) and subsequently found and highlighted by other scientific reports [54–58] as a relevant target in the pathogenesis processes of *P. salmonis.* T4SS is indeed an important virulence factor found in a variety of intracellular pathogens, including *Helicobacter pylori*, *Legionella pneumophila*, among others [19, 21, 34]. T4SS expression in virulent *P. salmonis* strains has been demonstrated using SHK-1 cell cultures, which mimic natural infection circumstances, or cell-free culture media with iron deprivation, through RNA-Seq or microarray approaches [54–56]. In those studies, T4SS expression was accompanied by a series of different flagella-structured genes, iron metabolism-related genes, oxidative stress, and drug resistance genes, among others, supporting the hypothesis that all of them are part of the adaptive response of the bacteria to stringent conditions and that they play an important role in the pathogenic process.

In another study, a genomic analysis of two virulent strains of the *P. salmonis* belonging to the EM90 and LF89 genogroups revealed that the first strain had two T4SS systems, whereas, the second strain had three. This is explained by 20-kb insertion/deletion (indel) in the LF89 genogroup strain containing the *tra* genes. Also, the authors speculate that those *indels* could be related to the differences in the range of hosts that both strains can affect [57].

Similarly, it was recently revealed that the region containing T4SS undergoes a genomic rearrangement within an attenuated *P. salmonis* strain, as a result of consecutive passages in a synthetic culture medium, which could be linked to the loss of virulence [59]. This genomic plasticity of *P. salmonis* is probably related to the differentiation and constant evolution of the two recognized genogroups (LF89 and EM90) described to date [38, 60], and closely related to the difference in virulence and resistance to antibiotics between the strains isolated by different research groups [2, 39, 61].

Many virulent strains of *Y. ruckeri* have the *tra* operon encoding T4SS, which has been suggested to be necessary for the pathogen survival in fish macrophages, and that showed similarity in sequence and organization to the *tra* operon of *Serratia entomophila*, with some of them showing sequence similarity to the Dot/Icm proteins of *Legionella pneumophil*n [16]*.* Moreover, a mutant *tra* strain was less virulent than the wild-type strain supporting its key role in pathogen survival in fish macrophages. Additionally, the expression of the *tra* genes in *Y. ruckery* is regulated by temperature and nutrient availability, showing its highest expression at 18 °C with nutrient limitation [16]. Previously it had been shown that the expression of the *traHIJKCLMN* cluster encoding the T4SS of *Y. ruckeri* was reduced by 64% upon incubation at 28 °C in comparison to 18 °C [62], which is very relevant since *Y. ruckeri* has an optimal growth temperature of 28 °C but the outbreaks occur at temperatures around 18 °C. Up-regulation of several genes related to *Y. ruckeri* pathogenesis has been observed at 18 °C instead of 28 °C [63] being a factor to consider in future studies.

The T4SS is very versatile, being able to secrete a series of effector molecules within the host, including DNA, proteins, protein complexes, and DNA–protein complexes, although their specific nature varies from bacteria to bacteria [64, 65]. The repertoire of effectors that are delivered to the cytosolic space is key in the pathogenesis process and the manipulation of the host's cellular physiology [64], but in general there are three major groups of T4SS involved in: DNA transfer (conjugative T4SSs), DNA uptake/release to and from the extracellular space, and transfer virulence factors and protein complexes into host cells [65]. Therefore, identifying them in each case is essential to elucidate whether they act on the modification of the cytoskeleton, and the modulation of vesicle traffic as *Salmonella,* or escapes from the phagosome blocking fusion with the lysosome such as *Francisella* or *Shigella* genres [31, 64] to mention a few examples.

A couple of good approximations to the proteomic identification of a set of virulence factors in *P. salmonis* were recently reported. The first of them characterized the protein profiles of the bacteria and the infected cell at two stages defined as “propagation” and “vacuolization.” In general, the proteins identified were classified within the following functional groups: cell motility, extracellular structures, intracellular traffic, vesicular secretion and transport, transport and metabolism of lipids, and prophages and transposons [66]. Among them, it was identified effector delivery systems such as T4SS, proteins related to flagella formation, toxins, and stress adaptation proteins, as well as iron capture systems, those that also present differences in expression between the different stages of the infection studied [66]. The second study was made with a highly virulent strain of *P. salmonis* (Austral005) and conducted to identify a protein profile that could be used as a vaccine formula (67). A set of 28 proteins were classified as virulence factors involved in adherence, efflux, iron metabolism, secretion systems, and stress response. Among them, the most relevant are *katA* (catalase), *sodCI* (a superoxidase dismutase precursor), *ahpC* (an alkyl hidroperoxidase), *bcfH* (a thiol-disulfide isomerase), and *ML1683* (a histone-like protein) all directly involved in bacterial stress response [18, 67]. Additionally, several virulence factors play a relevant role in the infection process, such as *pilA*-*pilC* involved in the formation of pili, or *fliE* related to the flagellum and *virB9-1*, *icmG* and *icmE*, genes belonging to the T4SS, which was already discussed in this manuscript as a key factor in the virulence process. Finally, the genes *fur* and *hasF* related to iron metabolism were also identified which gives rise to the discussion regarding the importance of this cofactor in the stress response and the pathogenesis of these bacterial pathogens.

Although numerous studies have shown the direct relationship of T4SS systems in the virulence and pathogenesis of various bacterial species, and as we have reviewed here several others are showing a relationship between oxidative stress and T4SS expression, there are still very few that show a direct relationship between the establishment of the response to the surrounding oxidative stress, T4SS expression and an increase in virulence or pathogenesis, which represents a clear challenge for the coming years of research.

## Iron metabolism and stress response

Iron is an essential nutrient for many cellular processes, being crucial in energy metabolism, virulence, and effective host infection, and as a cofactor for several enzymatic activities in bacterial pathogens [68]. However, both iron deficiency and iron overload can affect the redox state, hence the modulation of iron homeostasis is a critical process and bacteria have evolved multiple systems that contribute to its regulation.

Regarding the pathogenic process, the levels of free iron in the biological fluids within the host are extremely limited because the element is strongly bound to high-affinity iron-binding proteins. To obtain this scarce iron, most pathogenic bacteria have developed iron uptake systems that usually involve two components: (a) low-molecular-weight siderophores released by the bacteria that will chelate iron and subsequently transfer it to the pathogen and (b) iron-regulated outer membrane proteins (IROMPs) that function as receptors of the iron-siderophores complexes [43, 69]. Thus, during bacterial infection, when iron availability is limiting for bacterial growth, the siderophores produced by bacteria are secreted into the extracellular environment, where they form a siderophore-Fe^3+^complex, which is then captured by the bacterium through a specific bacterial outer membrane receptor. Once inside the bacterium, the Fe^3+^ is reduced to Fe^2+^ to release free iron, which can then be utilized in metabolic processes [68].

*Tenacibaculum spp* members can express high-affinity iron uptake mechanisms, which can compete with the host iron-binding proteins [43]. This bacterium produces bisucaberin B siderophores and transporters, heme-related proteins, iron-regulation proteins, and a Fur regulator homolog [44]. Avendaño-Herrera et al. (2005) described that an environmental strain of *T. maritimum* utilized several iron sources like hemoglobin, ferric ammonic citrate, hemin, and transferrin when added to iron-deficient media, allowing the bacteria the utilization of heme groups from the host as an iron source by direct binding [43].

Similarly, *R. salmoninarum* possesses iron‐acquisition mechanisms related to (a) NADPH reductase [70]; (b) siderophore production; and (c) haem utilization [71]. These bacteria have genes related to hemin uptake mechanisms, including, permeases, receptors, ATPase subunits, and haem oxygenases. Studies revealed that these genes are induced under iron-limited conditions, for iron acquisition purposes. In addition, the increased expression of these iron‐acquisition mechanisms by *R. salmoninarum* generates a hypervirulent phenotype, which proposes these genes as attractive candidates for therapeutic tools against *R. salmoninarum* [72].

For its part, *Y. ruckeri* produces the iron-chelating enterobactin-like siderophore ruckerbactin to capture iron [73]. The genes encoding the ruckerbactin are *rucC* and *rucD*, whose expression is upregulated under iron starvation and at 18 °C, but not at 28 °C [63], again interrelating the physiological condition with the virulence potential of this pathogen. Furthermore, *Y. ruckeri* possesses the *yhlBA* operon which contains the *yhlA* and *yhlB* genes [63] encoding a hemolysin and its activation and secretion partner respectively [74]. The increased expression of this operon is regulated by iron availability but also by temperature (18 °C) and leads to cytolysis and hemolysis of fish cells allowing it to capture the iron contained in them [74]. The *yhlBA* cluster is conserved among several *Y. ru*ckeri strains isolated from different sources and countries highlighting the relevance of this operon in *Y. ruckeri* virulence [73, 75].

Additionally, some proteomics analyses of *Y. ruckeri* strains grown under iron-limiting conditions and comparative bioinformatic analysis identified the participation of some outer membrane proteins (OMPs) related to iron transport (ShuA, HemR, and other TonB-dependent receptors). Their inherent role in iron homeostasis and their expression profiles under iron-poor conditions provide insights arguing for the potential roles of these proteins in host–pathogen interactions and *Y. ruckeri* virulence [76–78].

Concerning the response of *P. salmonis* against iron deprivation inside the host cell, the study of the expression patterns of the bacteria has led to the identification of several genes related to the iron capture and metabolism and also the regulator Fur [37, 41] which regulates the iron uptake but also the expression of several virulence factors in pathogen Gram-negative bacteria [18, 19, 34] and that was later functionally characterized [79]. Complementarily, in the iron stringent condition, *P. salmonis* showed the overexpression of RelA and SpoT, which are related to the synthesis and modulation of (p)ppGpp that in turn modulates the response to stress against amino acid starvation among others including iron [80]. Furthermore, it is known that (p)ppGpp modulates positively the RpoS transcriptional factor which commands the general stress response in bacteria including effectors and T4SS [18–21, 34, 81] that consequently presented an increase in expression of over eightfold during the intracellular growth of *P. salmonis* [80] which was observed also in late infection stages [66].

In a recent study, an iron chelator (e.g., DFP) was employed to highlight the role of iron availability in the infective process [82]. In general, the cytopathic effect was reduced in vitro using SHK-1 cells, and mortality was reduced in rainbow trout groups treated with two different concentrations of the chelator when compared to controls. Although the authors warn and discuss the need to make fine adjustments in the diets to determine the doses of use (due to the physiological imbalance that its use could cause), this approach opens up an excellent opportunity to characterize the stress response of *P. salmonis* and the other pathogens facing this new alternative.

## Biofilm formation

As previously stated, the interaction of these pathogens' molecular responses in the infection process is complex and involves multiple processes. In this regard, it is important to note that among the functions related to RelA-SpoT, (p)ppGpp, and RpoS is the activation of biofilm formation in various pathogenic bacteria facing stringent conditions [19], affecting the expression of genes related to virulence, toxin production, motility, and chemotaxis, among others, across a given bacterial pathogenic population, contributing to bacterial adaptation and colonization [19]. Furthermore, it has been reported that the activation of a two-component system known as UvrY/ BarA in E. coli occurs after the activation of RpoS, allowing the synthesis of many non-coding RNAs that influence the transcriptional response in the same way as Y. ruckeri [53, 55, 16].

Regarding the biofilm formation, some cultures of *P. salmonis* virulent strains belonging to LF89 and EM90 genogroups grown under different NaCl and Fe concentrations showed an increase in biofilm formation [83], evidencing the relationship between these stress conditions and the response. Furthermore, it was established that the planktonic forms of some strains of the EM genogroup and the sessile forms of some strains of the LF89 genogroup presented higher levels of virulence in SHK-1 cell cultures [83] which also could be related to the differences in virulence observed between strains of both genogroups [2, 39, 61]. In the same way, another study reports differences in the time and viability of biofilms formed in vitro by two different virulent isolates of *P. salmonis* (also belonging to LF89 and EM90 genogroups) independently of the nutritional content of the medium, but interestingly they showed differences in tolerance or sensitivity to salmon mucus, which is the first barrier of salmon, with the isolate belonging to genogroup EM 90 genogroup being more tolerant [84].

Biofilm formation in T. maritimum cells has been observed on hard surfaces [85], which may be related to their hydrophobicity and pathogenicity against turbot (Scophthalmus maximus), but it has also been observed that the bacteria are capable of producing substantial amounts of "slime", allowing them to adhere to hydrophobic surfaces, which may be related to their ability to adhere to the fish's external surfaces [86]. It is probable that the ability of *T. maritimum* to adhere would be related to pathogenicity and virulence since adhesive isolates of this bacteria appear to be more pathogenic than other less adherent [87]. In the same way, three different *T. dicentrarchi* strains were tested for their ability to adhere to an inert surface and to generate biofilms showing that all the strains were able to do so [88].

Also, *Y. ruckeri* can form biofilms in different materials found in fish farms, being a key factor in recurrent infections in rainbow trout aquaculture [16]. Recently the role of two membrane autotransporters called invasion Yrlnv and Invasine-like molecule Yrllm was investigated showing that both have a promoting effect on biofilm formation over different abiotic surfaces [89]. The biofilm formation over those surfaces gives *Y. ruckeri* a higher tolerance against antibiotics compared to planktonic cells due to the biofilm itself but also due to the change in the outer membrane pattern of the surface-associated cells of these virulent strains [16]. Additionally, this pathogen can adhere to, and invade various fish cell lines, a process that is closely related to the presence and expression of flagella in virulent strains, and with the quorum sensing system that regulates the expression of virulence factors, motility, and pathogen entry into host cells [16].

Inert surfaces in aquaculture environments can house biofilms and serve as reservoirs for pathogenic bacteria, demonstrating the importance of biofilms in disease transmission [16, 84–86].

## Conclusions

There are different levels of knowledge about the bacterial pathogens that affect aquaculture in Chile. However, there is considerable scientific evidence regarding the pathogenicity mechanisms used by different well-known pathogens and that—in general—present similar systems and strategies. The use of this comparative information can serve to advance more quickly in the identification and characterization of the different components that make up each one. In particular, the knowledge of the effectors that T4SS systems transport once inside macrophages, and which of them are common and/or different among the pathogens that affect salmonids, the understanding of the response mediated by iron starvation, or the regulation and blocking of biofilm formation in the surrounding structures, will give us insights on how to efficiently can control future infectious outbreaks, improving the advances already made and avoiding the excessive use of antibiotics to the different pathogens of the national aquaculture.

## Data Availability

Not applicable.

## Abbreviations

*S. salar*: *Salmo salar*
*O. mykiss*: *Oncorhynchus mykiss*
*O. kisutch*: *Oncorhynchus kisutch*
*R. salmoninarum*: *Renibacterium salmoninarum*
spp: *species*
P. salmonis: Piscirickettsia salmonis
SRS: Salmonid Rickettsial Septicemia
*Y. ruckeri*: *Yersinia ruckeri*
ERM: Enteric Redmouth Disease
RNS: Reactive Nitrogen Species
NO: Nitric Oxide
ROS: Reactive Oxygen Species
*T. maritimum*: Tenacibaculum maritimum
RNA: Ribonucleic acid
T4SS: Type IV Secretion System
LF89-EM90: *P. salmonis* genogroups
DNA: Desoxi Ribonucleic Acid
IROMPs: Iron-Regulated Outer Membrane Proteins
NADPH: Nicotinamide Adenine Dinucleotide Phosphate
ATP: Adenosine Triphosphate
OMP: Outer Membrane Proteins
ppGpp: Guanosine Pentaphosphate
SHK-1: Salmon Head Kidney -1 Cell Line
DFP: Deferiprone

## References

[1] Poblete EG, Drakeford BM, Ferreira FH, Barraza MG, Failler P (2019). The impact of trade and markets on Chilean Atlantic salmon farming. Aquacult Int.

[2] Maisey K, Montero R, Christodoulides M (2017). Vaccines for piscirickettsiosis (salmonid rickettsial septicaemia, SRS): the Chile perspective. Expert Rev Vaccines.

[3] Henríquez P, Kaiser M, Bohle H, Bustos P, Mancilla M (2016). Comprehensive antibiotic susceptibility profiling of Chilean *Piscirickettsia salmonis* field isolates. J Fish Dis.

[4] Avendaño-Herrera R, Collarte C, Saldarriaga-Córdoba M, Irgang R (2020). New salmonid hosts for *Tenacibaculum* species: expansion of tenacibaculosis in Chilean aquaculture. J Fish Dis.

[5] Rozas M, Enríquez R (2014). Piscirickettsiosis and *Piscirickettsia salmonis* in fish: a review. J Fish Dis.

[6] Suzuki M, Nakagawa Y, Harayama S, Yamamoto S (2001). Phylogenetic analysis and taxonomic study of marine Cytophaga-like bacteria: proposal for *Tenacibaculum* gen. nov. with *Tenacibaculum*
*maritimum* comb. nov. and *Tenacibaculum*
*ovolyticum* comb. nov., and description of *Tenacibaculum*
*mesophilum* sp. nov. and *Tenacibaculum*
*amylolyticum* sp. nov. Int J Syst Evol Microbiol.

[7] Avendaño-Herrera R, Toranzo AE, Magariños B (2006). Tenacibaculosis infection in marine fish caused by *Tenacibaculum maritimum*: a review. Dis Aquat Org.

[8] López JR, Piñeiro-Vidal M, García-Lamas N, de la Herran R, Navas JI, Hachero-Cruzado I, Santos Y (2010). First isolation of *Tenacibaculum*
*soleae* from diseased cultured wedge sole, *Dicologoglossa*
*cuneata* (Moreau), and brill, *Scophthalmus*
*rhombus* (L.). J Fish Dis.

[9] Avendaño-Herrera R (2016). Isolation, characterization and virulence potential of *Tenacibaculum dicentrarchi* in salmonid cultures in Chile. Transbound Emerg Dis.

[10] Irgang R, González-Luna R, Gutiérrez J, Poblete-Morales M, Rojas V, Tapia-Cammas D, Avendaño-Herrera R (2017). First identification and characterization of *Tenacibaculum dicentrarchi* isolated from Chilean red conger eel (*Genypterus chilensis*, Guichenot 1848). J Fish Dis.

[11] Hilger I, Ullrich S, Anders K (1991). A new ulcerative flexibacteriosis-like disease (‘yellow pest’) affecting young Atlantic cod *Gadus morhua* from the German Wadden Sea. Dis Aquat Org.

[12] Olsen AB, Gulla S, Steinum T, Colquhoun DJ, Nilsen HK, Duchaud E (2017). Multilocus sequence analysis reveals extensive genetic variety within *Tenacibaculum*
*spp*. associated with ulcers in sea-farmed fish in Norway. Vet Microbiol.

[13] Sanders JE, Fryer JL (1980). *Renibacterium*
*salmoninarum* gen. nov., sp. nov., the causative agent of bacterial kidney disease in salmonid fishes. Int J Syst Bacteriol.

[14] Iwama G, Nakanishi T (1997). The fish immune system: organism, pathogen, and environment.

[15] Kumar G, Menanteau-Ledouble S, Saleh M, El-Matbouli M (2015). *Yersinia ruckeri*, the causative agent of enteric redmouth disease in fish. Vet Res.

[16] Wrobel A, Leo JC, Linke D (2019). Overcoming fish defences: the virulence factors of *Yersinia ruckeri*. Genes.

[17] Mardones FO (2018). Identification of research gaps for highly infectious diseases in aquaculture: the case of the endemic *Piscirickettsia salmonis* in the Chilean salmon farming industry. Aquaculture.

[18] Kumar A, Rahal A, Sohal JS, Gupta VK (2021). Bacterial stress response: understanding the molecular mechanics to identify possible therapeutic targets. Expert Rev Anti Infect Ther.

[19] Trastoy R (2018). Mechanisms of bacterial tolerance and persistence in the gastrointestinal and respiratory environments. Clin Microbiol Rev.

[20] Giuliodori AM, Gualerzi CO, Soto S, Vila J, Tavío MM (2007). Review on bacterial stress topics. Ann NY Acad Sci.

[21] Grant SS, Hung DT (2013). Persistent bacterial infections, antibiotic tolerance, and the oxidative stress response. Virulence.

[22] McCarthy UM (2008). Survival and replication of *Piscirickettsia salmonis* in rainbow trout head kidney macrophages. Fish Shellfish Immunol.

[23] Rojas V, Galanti N, Bols NC, Jiménez V, Paredes R, Marshall SH (2010). Piscirickettsia salmonis induces apoptosis in macrophages and monocyte-like cells from rainbow trout. J Cell Biochem.

[24] Rojas V, Galanti N, Bols NC, Marshall SH (2009). Productive infection of *Piscirickettsia salmonis* in macrophages and monocyte-like cells from rainbow trout, a possible survival strategy. J Cell Biochem.

[25] Ramírez R, Gómez FA, Marshall SH (2015). The infection process of *Piscirickettsia salmonis* in fish macrophages is dependent upon interaction with host-cell clathrin and actin. FEMS Microbiol Lett.

[26] Ryckaert J, Bossier P, D’Herde K, Diez-Fraile A, Sorgeloos P, Haesebrouck F, Pasmans F (2010). Persistence of *Yersinia ruckeri* in trout macrophages. Fish Shellfish Immunol.

[27] Bandin I, Ellis AE, Barja JL, Secombes CJ (1993). Interaction between rainbow trout macrophages and *Renibacterium salmoninarum in vitro*. Fish Shellfish Immunol.

[28] Gutenberger SK, Duimstra J, Rohovec J, Fryer J (1997). Intracellular survival of *Renibacterium salmoninarum* in trout mononuclear phagocytes. Dis Aquat Org.

[29] Sudheesh PS, Crane S, Cain KD, Strom MS (2007). Sortase inhibitor phenyl vinyl sulfone inhibits *Renibacterium salmoninarum* adherence and invasion of host cells. Dis Aquat Org.

[30] Campos-Pérez JJ, Ellis AE, Secombes CJ (2000). Toxicity of nitric oxide and peroxynitrite to bacterial pathogens of fish. Dis Aquat Org.

[31] Diacovich L, Gorvel JP (2010). Bacterial manipulation of innate immunity to promote infection. Nat Rev Microbiol.

[32] Ma Z, Russo VC, Rabadi SM, Jen Y, Catlett SV, Bakshi CS, Malik M (2016). Elucidation of a mechanism of oxidative stress regulation in *Francisella tularensis* live vaccine strain. Mol Microbiol.

[33] Ma Z (2019). Stringent response governs the oxidative stress resistance and virulence of *Francisella tularensis*. PLoS ONE.

[34] Gottesman S (2017). Stress reduction, bacterial style. J Bacteriol.

[35] Gómez FA, Cárdenas C, Henríquez V, Marshall SH (2011). Characterization of a functional toxin-antitoxin module in the genome of the fish pathogen *Piscirickettsia salmonis*. FEMS Microbiol Lett.

[36] Gómez FA, Tobar JA, Henríquez V, Sola M, Altamirano C, Marshall SH (2013). Evidence of the presence of a functional Dot/Icm type IV-B secretion system in the fish bacterial pathogen *Piscirickettsia salmonis*. PLoS ONE.

[37] Pulgar R, Travisany D, Zuñiga A, Maass A, Cambiazo V (2015). Complete genome sequence of *Piscirickettsia salmonis* LF-89 (ATCC VR-1361) a major pathogen of farmed salmonid fish. J Biotechnol.

[38] Nourdin-Galindo G (2017). Comparative pan-genome analysis of *Piscirickettsia salmonis* reveals genomic divergences within genogroups. Front Cell Infect Microbiol.

[39] Lagos F, Cartes C, Vera T, Haussmann D, Figueroa J (2017). Identification of genomic islands in Chilean *Piscirickettsia salmonis* strains and analysis of gene expression involved in virulence. J Fish Dis.

[40] Bohle H, Henríquez P, Grothusen H, Navas E, Bustamante F, Bustos P, Mancilla M (2017). The genome sequence of an oxytetracycline-resistant isolate of the fish pathogen *Piscirickettsia salmonis* harbors a multidrug resistance plasmid. Genome Announc.

[41] Yañez AJ (2014). Draft genome sequence of virulent strain AUSTRAL-005 of *Piscirickettsia salmonis*, the etiological agent of piscirickettsiosis. Genome Announc.

[42] Sheng Y, Abreu IA, Cabelli DE, Maroney MJ, Miller AF, Teixeira M, Valentine JS (2014). Superoxide dismutases and superoxide reductases. Chem Rev.

[43] Avendaño-Herrera R, Toranzo AE, Romalde JL, Lemos ML, Magariños B (2005). Iron uptake mechanisms in the fish pathogen *Tenacibaculum maritimum*. Appl Environ Microbiol.

[44] Pérez-Pascual D (2017). The complete genome sequence of the fish pathogen *Tenacibaculum maritimum* provides insights into virulence mechanisms. Front Microbiol.

[45] Lee M, Jun SY, Yoon BY, Song S, Lee K, Ha NC (2012). Membrane fusion proteins of type I secretion system and tripartite efflux pumps share a binding motif for TolC in gram-negative bacteria. PLoS ONE.

[46] Bethke J, Yáñez AJ, Avendaño-Herrera R (2018). Comparative genomic analysis of two Chilean *Renibacterium salmoninarum* isolates and the type strain ATCC 33209T. Genome Biol Evol.

[47] Wiens GD, Dale OB (2009). *Renibacterium salmoninarum* p57 antigenic variation is restricted in geographic distribution and correlated with genomic markers. Dis Aquat Org.

[48] Densmore CL, Smith SA, Holladay SD (1998). *In vitro* effects of the extracellular protein of *Renibacterium salmoninarum* on phagocyte function in brook trout (Salvelinus fontinalis). Vet Immunol Immunopathol.

[49] Bruno DW (1998). The relationship between agglutination, cell surface hydrophobicity and virulence of the fish pathogen *Renibacterium*
*salmoninarum*. FEMS Microbiol Lett.

[50] Bruno DW (1990). Presence of a saline extractable protein associated with virulent strains of the fish pathogen *Renibacterium salmoninarum*. Bull Eur Assoc Fish Pathol.

[51] Kumar G (2016). Shotgun proteomic analysis of *Yersinia ruckeri* strains under normal and iron-limited conditions. Vet Res.

[52] Acuña LG (2021). Participation of two sRNA RyhB homologs from the fish pathogen *Yersinia ruckeri* in bacterial physiology. Microbiol Res.

[53] Segovia C, Arias-Carrasco R, Yañez AJ, Maracaja-Coutinho V, Santander J (2018). Core non-coding RNAs of *Piscirickettsia salmonis*. PLoS ONE.

[54] Millar AD, Tapia P, Gómez FA, Marshall SH, Fuentes DE, Valdes JH (2018). Draft genomes and reference transcriptomes extend the coding potential of the fish pathogen *Piscirickettsia salmonis*. Electron J Biotechnol.

[55] Zuñiga A (2019). Transcriptomic changes of *Piscirickettsia salmonis* During intracellular growth in a salmon macrophage-like cell line. Front Cell Infect Microbiol.

[56] Bohle Machuca A, Martinez V (2016). Transcriptome analysis of the intracellular facultative pathogen *Piscirickettsia salmonis*: Expression of putative groups of genes associated with virulence and iron metabolism. PLoS ONE.

[57] Bohle H (2014). Comparative genome analysis of two isolates of the fish pathogen *Piscirickettsia salmonis* from different hosts reveals major differences in virulence-associated secretion systems. Genome Announc.

[58] Valenzuela-Miranda D, Gallardo-Escárate C (2018). Dual RNA-Seq Uncovers metabolic amino acids dependency of the intracellular bacterium *Piscirickettsia salmonis* infecting Atlantic salmon. Front Microbiol.

[59] Valenzuela-Miranda D, Valenzuela-Muñoz V, Núñez-Acuña G, Gallardo-Escárate C (2020). Long-term serial culture of *Piscirickettsia salmonis* leads to a genomic and transcriptomic reorganization affecting bacterial virulence. Aquaculture.

[60] Otterlei A (2016). Phenotypic and genetic characterization of *Piscirickettsia salmonis* from Chilean and Canadian salmonids. BMC Vet Res.

[61] Ortiz-Severín J, Travisany D, Maass A, Chávez FP, Cambiazo V (2019). *Piscirickettsia salmonis* cryptic plasmids: source of mobile DNA and virulence factors. Pathogens.

[62] Méndez J, Fernández L, Menéndez A, Reimundo P, Pérez-Pascual D, Navais R, Guijarro JA (2009). A chromosomally located *traHIJKCLMN* operon encoding a putative type IV secretion system is involved in the virulence of *Yersinia ruckeri*. Appl Environ Microbiol.

[63] Fernández L, Márquez I, Guijarro JA (2004). Identification of specific *in vivo*-induced (ivi) genes in *Yersinia ruckeri* and analysis of ruckerbactin, a catecholate siderophore iron acquisition system. Appl Environ Microbiol.

[64] Voth DE, Broederdorf LJ, Graham JG (2012). Bacterial Type IV secretion systems: versatile virulence machines. Future Microbiol.

[65] Alvarez-Martinez CE, Christie PJ (2009). Biological diversity of prokaryotic type IV secretion systems. Microbiol Mol Biol Rev.

[66] Ortiz-Severín J, Travisany D, Maass A, Cambiazo V, Chávez FP (2020). Global proteomic profiling of *Piscirickettsia salmonis* and salmon macrophage-like cells during intracellular infection. Microorganisms.

[67] Pontigo JP (2021). Protein-based vaccine protect against *Piscirickettsia salmonis* in Atlantic salmon (*Salmo salar*). Front Immunol.

[68] Miethke M, Marahiel MA (2007). Siderophore-based iron acquisition and pathogen control. Microbiol Mol Biol Rev.

[69] Hood MI, Skaar EP (2012). Nutritional immunity: Transition metals at the pathogen-host interface. Nat Rev Microbiol.

[70] Grayson TH, Bruno D, Evenden A, Gilpin M, Munn C (1995). Iron acquisition by *Renibacterium salmoninarum*: contribution of iron reductase. Dis Aquat Org.

[71] Bethke J, Poblete-Morales M, Irgang R, Yáñez A, Avendaño-Herrera R (2016). Iron acquisition and siderophore production in the fish pathogen *Renibacterium salmoninarum*. J Fish Dis.

[72] Bethke J, Arias-Muñoz E, Yáñez A, Avendaño-Herrera R (2019). *Renibacterium salmoninarum* iron-acquisition mechanisms and ASK cell line infection: virulence and immune response. J Fish Dis.

[73] Tobback E, Decostere A, Hermans K, Haesebrouck F, Chiers K (2007). *Yersinia ruckeri* infections in salmonid fish. J Fish Dis.

[74] Fernández L, Prieto M, Guijarro JA (2007). The iron- and temperature-regulated haemolysin YhlA is a virulence factor of *Yersinia ruckeri*. Microbiology.

[75] Guijarro JA, García-Torrico AI, Cascales D, Méndez J (2018). The infection process of *Yersinia ruckeri*: reviewing the pieces of the jigsaw puzzle. Front Cell Infect Microbiol.

[76] Davies RL (1991). Outer membrane protein profiles of *Yersinia ruckeri*. Vet Microbiol.

[77] Romalde JL, Conchas RF, Toranzo AE (1991). Evidence that *Yersinia ruckeri* possesses a high affinity iron uptake system. FEMS Microbiol Lett.

[78] Ormsby MJ, Grahame E, Burchmore R, Davies RL (2019). Comparative bioinformatic and proteomic approaches to evaluate the outer membrane proteome of the fish pathogen *Yersinia ruckeri*. J Proteomics.

[79] Almarza O (2016). A functional ferric uptake regulator (Fur) protein in the fish pathogen *Piscirickettsia salmonis*. Int Microbiol.

[80] Zuñiga A (2019). Transcriptomic changes of *Piscirickettsia salmonis* During intracellular growth in a salmon macrophage-like cell line. Front Cell Infect Microbiol.

[81] Gollan B, Grabe G, Michaux C, Helaine S (2019). Bacterial persisters and infection: past, present, and progressing. Annu Rev Microbiol.

[82] Caruffo M (2020). Pharmacological iron-chelation as an assisted nutritional immunity strategy against *Piscirickettsia salmonis* infection. Vet Res.

[83] Santibañez N (2020). Biofilm produced in vitro by *Piscirickettsia salmonis* generates differential cytotoxicity levels and expression patterns of immune genes in the Atlantic salmon cell line SHK-1. Microorganisms.

[84] Levipan HA, Irgang R, Yañez A, Avendaño-Herrera R (2020). Improved understanding of biofilm development by *Piscirickettsia salmonis* reveals potential risks for the persistence and dissemination of piscirickettsiosis. Sci Rep.

[85] Levipan HA, Tapia-Cammas D, Molina V, Irgang R, Toranzo AE, Magariños B, Avendaño-Herrera R (2019). Biofilm development and cell viability: an undervalued mechanism in the persistence of the fish pathogen *Tenacibaculum maritimum*. Aquaculture.

[86] Avendaño-Herrera R, Toranzo AE, Magariños B (2006). Tenacibaculosis infection in marine fish caused by *Tenacibaculum maritimum*: a review. Dis Aquat Org.

[87] Van Gelderen R, Carson J, Gudkovs N, Nowak B (2010). Physical characterisation of *Tenacibaculum maritimum* for vaccine development. J Appl Microbiol.

[88] Levipan HA, Irgang R, Tapia-Cammas D, Avendaño-Herrera R (2019). A high-throughput analysis of biofilm formation by the fish pathogen *Tenacibaculum dicentrarchi*. J Fish Dis.

[89] Wrobel A (2020). The inverse autotransporters of *Yersinia ruckeri*, YrInv and YrIlm, contribute to biofilm formation and virulence. Environ Microbiol.

